# Adolescent health in the Covid-19 pandemic: a construction through Nola Pender’s model

**DOI:** 10.1590/0034-7167-2021-0696

**Published:** 2022-07-29

**Authors:** Daniela Bulcão Santi, Roberta Rossa, Luciene da Silva Santos Bomfim, Alcione Ribeiro Dias, Ieda Harumi Higarashi, Vanessa Denardi Antoniassi Baldissera

**Affiliations:** I Universidade Estadual de Maringá. Maringá, Paraná, Brazil; II Universidade Federal de Mato Grosso do Sul. Campo Grande, Mato Grosso do Sul, Brazil

**Keywords:** Adolescent Health, Health Promotion, COVID-19, Nursing Theory, Psychodrama, Salud del Adolescente, Promoción de la Salud, COVID-19, Teoría de Enfermería, Psicodrama, Saúde do Adolescente, Promoção da Saúde, COVID-19, Teoria de Enfermagem, Psicodrama

## Abstract

**Objective:**

To construct perspectives of adolescent health in the face of the Covid-19 pandemic, through emancipatory dialogues guided by Nola Pender’s Model.

**Method:**

This is a participatory research based on the methodology of Psychodramatic Pedagogy of María Alicia Romaña. The action was carried out with 17 school-aged adolescents from a city in the Center-West Region of Brazil. The theoretical-analytical framework used was Nola Pender’s Health Promotion Model, and the data collected were discussed based on Paulo Freire’s dialogical framework.

**Results:**

The workshop allowed the sharing of individual characteristics and experiences of adolescents regarding the reality experienced in the pandemic, highlighting mental health as a main theme for health promotion.

**Final considerations:**

It was construct, along with the adolescents, important knowledge about health in the pandemic, supporting the thematic reflection and the elaboration of timely health promotion interventions.

## RESUMO

**Objetivo:**

Construir perspectivas de saúde dos adolescentes em face à pandemia de Covid-19, a partir dos diálogos emancipatórios, orientados pelo modelo de Nola Pender.

**Método:**

Trata-se de uma pesquisa participativa, metodologicamente estruturada com a Pedagogia Psicodramática de María Alicia Romaña, realizada com 17 adolescentes escolares de um município na região Centro-Oeste do Brasil. Utilizou-se como referencial teórico-analítico o Modelo de Promoção da Saúde de Nola Pender, sendo os dados discutidos com base no referencial dialógico de Paulo Freire.

**Resultados:**

A oficina propiciou o compartilhamento de características e experiências individuais dos adolescentes sobre a realidade vivenciada na pandemia, evidenciando a saúde mental como tema protagônico para a promoção da saúde.

**Considerações finais:**

Foi possível elaborar com os adolescentes um conhecimento sobre a saúde na pandemia, fundamentando a reflexão temática e a construção de intervenções oportunas de promoção da saúde.

## RESUMEN

**Objetivo:**

Construir perspectivas de salud para adolescentes ante la pandemia de Covid-19, a partir de diálogos emancipatorios orientados por el modelo de Nola Pender.

**Método:**

Se trata de una investigación participativa, estructurada metodológicamente con la Pedagogía Psicodramática de María Alicia Romaña y llevada a cabo entre 17 adolescentes escolares en un municipio de la región Centro-Oeste de Brasil. Se utilizó el Modelo de Promoción de la Salud de Nola Pender como referencial teórico-analítico y los datos se plantearon con base en el referencial dialógico de Paulo Freire.

**Resultados:**

El taller propició que los adolescentes compartieran sus características y sus experiencias personales sobre la realidad vivida durante la pandemia, destacándose la salud mental como tema principal para la promoción de la salud.

**Consideraciones finales:**

Fue posible elaborar con los adolescentes un conocimiento sobre la salud en la pandemia, basado en la reflexión temática y en la construcción de intervenciones oportunas para la promoción de la salud.

## INTRODUCTION

In the Covid-19 pandemic caused by the new coronavirus (Covid-19) in 2020, children and adolescents represented a small proportion of cases and deaths reported in the world^(1)^.

In Brazil, more than 123 million people live with at least one person aged less than 17 years, that is, a person of school age^(2)^. In this context, the need for social isolation to avoid the transmission of the virus led adolescents to experience lack of social interaction, socioeconomic disparities, domestic violence, sedentary lifestyle, among others^(3)^.

The “*ConVid* Adolescents - Behavioral research” was a national survey that aimed to verify how the pandemic affected/changed the lives of adolescents. The results, referring to a sample of almost 10 thousand adolescents, showed important impacts on social relationships, school activities, health care and the mood of Brazilian adolescents during the period of social isolation^(4-5)^.

In this scenario of imminent danger of direct or indirect illness, reflections about health practices are relevant, so that health promotion can favor the population’s health care, social cohesion and collective responsibility for the health and well-being of the population ^(6)^.

In this context, nurses’ participatory educational practices, also considered care measures, have the potential to carry out the health promotion of adolescents, which must be based on autonomy. Learning processes that involve the transformation of values and lifestyles and the emancipation of communities are long-term processes that promote critical thinking, construction of transdisciplinary knowledge and transformative experiences^(7)^.

The Health Promotion Model (HPM) developed by Nola Pender enables nurses to develop care strategies, allowing professionals to plan, intervene and evaluate their activities according to three components: 1. Individual characteristics and experiences; 2. Behavior-specific cognitions and affect; and 3. Desired health promotion behavior^(8)^.

Adolescents were an important focus of interest in Nola Pender’s research and, currently, the HPM is used internationally in research, education and care practice with the most diverse populations. However, there is a gap in studies that use the HPM in participatory strategies, which can enhance health promotion, as it promotes emancipatory educational practices^9)^.

The object of this study was the use of the HPM with participatory strategies for the promotion of adolescent health, having the first component of the model as a guiding question, specifically “What individual characteristics and experiences of adolescents during the pandemic can be discussed in order to build emancipatory knowledge with their peers?”

## OBJECTIVES

To construct perspectives of adolescent health in the face of the Covid-19 pandemic, through emancipatory dialogues guided by Nola Pender’s Model.

## METHODS

### Ethical aspects

The project was approved by the Research Ethics Committee and Certificate of Presentation for Ethical Assessment (CAAE). Parents or guardians were asked to fill in the Informed Consent Term (TCLE) and the adolescents signed an Informed Agreement Term (TALE).

Resolutions No. 466/2012 and 510/2016 of the National Health Council were followed. To guarantee safety in the production and storage of data collected via technology, all the instructions provided in the document “Guidelines for procedures in research with any stage in virtual environment” of the National Research Ethics Commission (CONEP) were followed^(10)^.

To preserve anonymity, the participants were identified by codes, with the letter “E” for student, followed by the Arabic number corresponding to the order of their speech in the workshop, such as: E1, E2, E3, and so on.

### Theoretical and methodological framework and type of study

This is an action-research study with a participatory approach, which implies cooperation between the actors (researchers and participants) on a collective problem^(11)^. Action-research can be carried out through group activities, games and theater, used as technical resource to encourage dialogue and engagement of participants in the theme, allowing greater inclusion and participation and leading to the emancipation of knowledge through reflective practice ^(11-12)^.

The central theme considered as a collective problem was health promotion and, therefore, the theoretical and analytical framework for intervention was Nola Pender’s Health Promotion Model (HPM), especially the first component “Characteristics and individual experiences”, which includes Personal Factors (biological, psychological and sociocultural) and Prior Behavior ^(8)^. This component was chosen because it can guide the situational analysis of the participants’ health through the understanding of their health needs and the experiences that affect subsequent actions, helping to define target behavior.

As the differential of this research is the use of the HPM in a collective and participatory manner, and considering that both action-research method and the HPM do not require specific methods and techniques, Psychodramatic Pedagogy was chosen as a methodological reference to structure the execution of the workshop. This approach provides a practical, spontaneous and reflective method to allow the emergence of the main theme.

Psychodramatic Pedagogy is a method of research and intervention proposed by the Argentinian educator María Alicia Romaña and based on the thoughts of Jacob Levy Moreno, Paulo Freire and Vigotski. The author proposes three stages to carry out the workshop: (1) unspecific and specific warm-up, (2) dramatic action and (3) sharing^(13)^.

Therefore, active and mutual participation was promoted during the workshop, which was based on the first component of the HPM and structured according to Psychodramatic Pedagogy, favoring the collective construction of knowledge and raising awareness on health promotion among adolescents in the context of the pandemic.

### Study setting

The research site was a federal rural education institution in a city in the Center-West Region of Brazil.

### Data source

Twenty-five high school adolescents enrolled in the technical course were invited to participate in the study. The inclusion criteria were: being in the second year of high school and having availability and access to the internet. The exclusion criteria were: students who were away during data collection. Parents or guardians and the adolescents were contacted individually by *Whatsapp®*. A total of 17 adolescents participated in the research.

The group of researchers was composed of two nurses who are doctoral students in nursing, in partnership with a psychologist and a Literature teacher with expertise in psychodrama. One of the nurses and the teacher worked at the institution and had a previous bond with the participants, established in other activities and care.

### Data collection and organization

Data was collected virtually in February 2021, through a *Google Forms®* questionnaire and a workshop held on the *Zoom®* platform, enabling the elaboration of individual and collective perspectives on health in the period of social distancing imposed by Covid-19.

**Figure 1:**
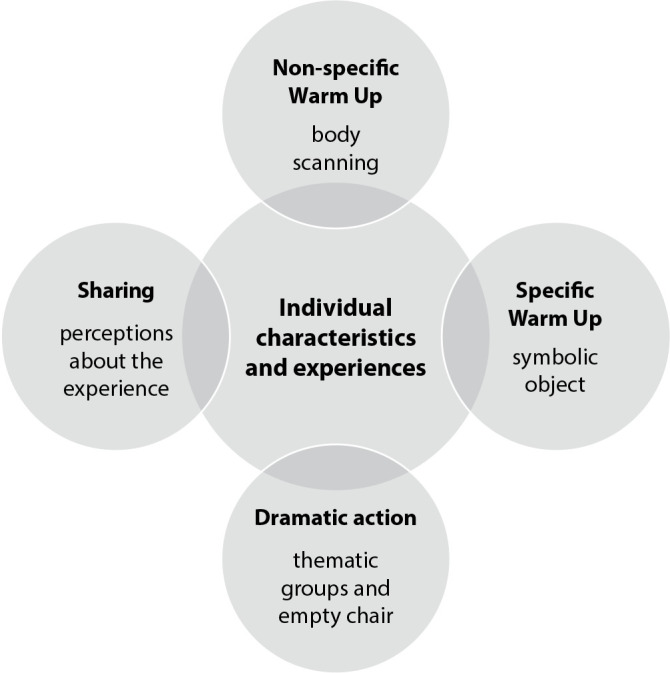
Methodological course of action research mediated by Psychodramatic Pedagogy for the promotion of adolescent health in the context of the Covid-19 pandemic, Brazil, 2021

Considering the first component of the HPM, a questionnaire with 24 questions was elaborated and applied by the researchers with the purpose of gathering information about Personal Factors (biological, psychological and sociocultural factors). During the workshop, the researchers tried to incorporate and work with the information on Previous Behavior obtained. The steps of the Psychodramatic Pedagogy method ^(13)^ were followed in the execution of the workshop and are described below and included in Figure 1:

- For the non-specific warm-up, the group activity proposed was a body scanning technique, a type of guided relaxation with music that guides attention to the parts of the body;
- The specific warm-up was a game with a symbolic object, in which each student should choose and present an object that represented their health during the pandemic;
- For the dramatic action, the teenagers were divided into groups according to one of the themes presented: “Healthy life is everything” or “My lifestyle is limitless”. They discussed the topic and, at the end of that moment, they were instructed to imagine themselves as candidates for Minister of Health and propose a campaign theme - the proposal was presented using an empty chair, as if it were the minister’s chair;
- For the sharing moment, the adolescents were encouraged to present what was most significant for them in the workshop experience.

### Data analysis

The virtual workshop lasted 2 hours and 30 minutes and was recorded and transcribed. Later, using this material and the answers to the questionnaire, an interpretative analysis was carried out, seeking shared perceptions that corresponded to the themes of the first component of Nola Pender’s HPM, namely: Personal Factors (biological, psychological and sociocultural) and Prior Behavior^(8)^. As this process provided critical and reflective dialogues, the discussion was based on the dialogic framework of Paulo Freire ^(12)^, in an attempt to understand the emancipatory outcomes of this path, with a focus on the problem of health promotion.

The elaboration of this manuscript followed the items for qualitative studies provided by the *Consolidated criteria for reporting qualitative research* (COREQ)^(14)^.

## RESULTS

The questionnaire filled in by the students allowed the understanding of individual data and singularities. In the virtual workshop, it was possible to (re)cognize the characteristics and experiences in the context of the pandemic in a collective perspective, according to the experiences shared and the health perspectives constructed. Following the HPM, these factors will be listed in the following categories: Personal Factors (biological, psychological and sociocultural) and Previous Behavior.

### Personal factors: Biological, psychological and sociocultural

The HPM clarifies that these factors can be either inherited or acquired^(8)^. Thus, the individual questionnaire was essential for a detailed evaluation of the participants. Data regarding the personal factors investigated are presented in Table 1.

**Table 1:** Personal, biological and sociocultural factors of students from a federal public institution in a city in the Center-West Region, Brazil, 2021

STUDENTS	(N=17)
BIOLOGICAL FACTORS	
Gender	
Male	09
Female	08
Age	
16 years	09
17 years	07
18 years	01
Ethnicity	
White	09
Black	07
Indigenous	01
Is in a risk group for Covid-19^*^	
No	17
Has had a diagnosis or suspicion of Covid-19	
No	13
How they evaluate their health before the pandemic	
Good or great	13
Perception of weight change during the pandemic	
Weight gain	07
Weight loss	04
Class of foods most often consumed	
Processed	08
In natura	07
Ultra-processed	02
Daily water consumption	
Between 1 and 2 liters	10
Knowledge about their vaccination status	
Up to date	13
Frequency of medical evaluation	
Only when they get sick	08
Annually, for routine evaluation	08
Regularly, in follow-up	01
**SOCIO-CULTURAL FACTORS**	
Religion/belief	
Christian	12
Number of people living in their household	
4 or more people	09
Lives with a person in a risk group for Covid-19^*^	
Yes	09
Diagnosis or suspicion of Covid-19 in a person from their household	
No	12
Screen time	
More than 4 hours	13
Has good internet connection for remote activities	
Yes	13
Has a computer to access remote activities	
Yes	16
Has a paid job	
No	14

* *Note: Risk factors for Covid-19: being 60 years old or older, brain, heart, lung, liver or kidney disease in severe or decompensated state, hypertension, diabetes, immunosuppression, cancer, morbid obesity and being pregnant or breastfeeding (Covid-19 Epidemiological Surveillance Guide - version 3, Ministry of Health, 2021).*

The biological factors were similar for both genders, with a predominance of older adolescents (16-18 years old), of white or mixed ethnicity. All of them reported not being in any risk group and most had no confirmed or suspected diagnosis of Covid-19 (n=13). Regarding health care, they had knowledge about their vaccination status (n=13) and attended medical evaluations anually (n=8). Data on weight changes and diet with a significant number of processed foods (n=8) are highlighted.

In the virtual workshop, the physical repercussions of a more inactive routine and the lack of attention to the body were confirmed.

*I became sedentary in this quarantine; I feel pain everywhere.* (E8)

*Sometimes our minds are focused on so many other things that we don’t even remember that we have a body to take care of, and we also just do the normal things of our routine and forget about our own body, you know?!* (E3)

As for psychological factors, the students answered open questions in the questionnaire about self-esteem, motivation, sleep and mental health care. The responses show significant changes, and the reasons pointed out were the uncertainties caused by the pandemic and the lack of a routine.

*At first, it was kind of boring to sit still* [...] *when our classes became virtual, I started to sleep later, as I didn’t have to go to school.* (E5)

*Before the pandemic, my sleep was well-regulated. Now I sleep very late and wake up very late.* (E14)

*In the beginning, I couldn’t sleep for some nights because I was very worried and afraid. It took me a while to get used to the whole situation, and sometimes I still have trouble to sleep, even when I’m tired.* (E11)

As strategies for mental health care, students cited reading activities, games, watching series, dialogue, meditation, crafting and physical activity. To understand the feelings experienced, one of the questions asked them to select the feelings with which they identified the most. This list of feelings was based on the Non-Violent Communication framework, a light care technology that allows recognizing human needs that are met or not^(15)^. Most students (n=11) indicated feelings related to the needs met (calm, happy, hopeful, satisfied) and six only marked those related to unmet needs (sad, anxious, exhausted, alone).

In the virtual workshop, the psychological effects of this routine were confirmed in reports of experiences of extreme feelings.

*I felt the energy to do things, to be with people, but I couldn’t, I Was hyperactive.* (E10)

*Sometimes I’m agitated, with nothing to do, and sometimes I start to think useless thing.s* (E12)

*Even though we went through difficult times, it is passing, I believe that the battle is not lost.* (E6)

*There are some feelings running through my head, I feel like there is a void. That agitation in my life is over, it’s completely over. I used to do a lot of things, now I just do a few things. And that affects me, it gives me bad vibes.* (E2)

*We are living through a very complicated period and our mental health is a turmoil.* (E13)

*We get a little confused at this moment, like I said before, in this matter of mental health, we get a little crazy. It seems like it’s just us.* (E3)

Regarding sociocultural factors related to Covid-19, none of the students reported being in a risk group, but most of them (n=09) reported living with someone at risk of severe illness. In the virtual workshop, students shared the challenges of social isolation that required a new routine at home, which included: the organization of time, family living and the restriction of social contacts, as shown in the following speeches:

*Inside the house, I realized that time does not pass as fast as I expected, it is too slow.* (E16)

*Time passed too slow, I didn’t know what to do, I had no one to talk to, I felt very lonely.* (E15)

*It was very difficult to be alone at home and, obviously, I had my parents, but it is not the same thing as having friends around.* (E1)

*I was tucked away in this quarantine, quiet in my house, just tucked away.* (E13)

*We are distant, each one in their own house, and especially my father, because now he is not at home and a lot happened at the end of the year.* (E11)

*Whenever I’m feeling down like that, my brother is by my side, always.* (E9)

Students expressed feelings of frustration and concern regarding online school activities, since, in their perception, presence at school is important for effective learning.

*I think that the most difficult thing was my learning at school, because for me, I am a person who has difficulties, I need to be there, paying attention, I need help, and I think that time passed too fast, I couldn’t learn as much as I would have learned at school.* (E14)

*So much happened and, at the same time, we were just standing still, you know?!* (E3)

### Prior behavior

In the HPM, this component proposes a situational analysis of habits and living conditions^(8)^. It was observed, mainly with the intermediate object technique, that students relate health perceptions with entertainment practices that help them in the challenges experienced during social isolation.

*I like to listen to music, I listen to it all day. I think music is great, it’s calming or sometimes it cheers me up, it depends on the music.* (E8)

*Every time I put on an upbeat song, I felt energized, regardless of what was happening around me.* (E7)

*Music calms me down, helps me relax and then I can think better.* (E12)

*Watching Formula 1, it’s a sport that my father likes, and every Sunday morning we religiously watch Formula 1 together, because it was a way for us to escape reality. It helped me.* (E1)

*Playing games with my friends was how I spent time.* (E5)

*Making crafts was something that distracted me a lot* [...] *and the cell phone, because it helps me get closer to people, since we are so far away.* (E11)

The speeches during the debate, mainly in the group “My lifestyle is limitless”, the period before the pandemic was characterized as a reference of life enjoyment and the experience of the pandemic, with the notion that it was (un)limited, was used as justification in the construction of supposedly reckless proposals.

*Before the pandemic, we were living the good life and we didn’t know. We were much happier than now that we can’t go out. So now we keep thinking that we should have enjoyed it more, and now there’s not much... there’s not much for us to enjoy anymore... you know?!* [laughs]. (E14)

*We have to enjoy life now so we don’t regret it later... we should not put off what we can do bow* [laughs]. (E3)

*We enjoy what we have now, what we have today, because tomorrow is uncertain, right?! So we live everything very intensely, because we don’t know what may or may not happen tomorrow.* (E12)

Aiming to apprehend the students’ perspectives in the experienced scenario, the empty chair technique was proposed, in which each student proposed a theme for their candidacy as Minister of Health. Vaccination and attention to quality of life were highlighted.

*Vaccines for all and lives above money.* (E12)

*Get vaccinated, protect who you love.* (E11)

*Enjoy and live intensely to make your time worthwhile and be no longer just a number.* (E3)

*Do whatever you like and enjoy today.* (E13)

*Be sure to spread love, time is short*. (E14)

*Live intensely, but responsibly.* (E16)

*The important thing is not how many years in your life, but how much health in your years.* (E15)

## DISCUSSION

The analysis of adolescent health using the HPM was fruitful, mainly because the use of participatory strategies allows understanding the meaning and impact of the experiences lived during the pandemic, demonstrated quantitatively in the “ConVid Adolescents” survey, from the perspective of the adolescents themselves^(4)^. The intervention via questionnaire and workshop was comprehensive and complementary, considering that for the promotion of community health, change must occur at different levels, starting with individuals and moving up to the community as a whole^(8)^.

As for biological Personal Factors, the reported perception of weight change and the increased frequency of consumption of processed foods were significant factors, added to the reports regarding the sedentary lifestyle of the participants in the workshop. The findings of the study corroborate a survey that compared habits before and during the pandemic and showed an increase in the consumption of frozen food, chocolate and sweets and in screen time, as well as a consequent decrease in physical activity^(4)^.

The lack of a routine, pointed out by the adolescents, occurs because they do not have to go to school, and this interferes with the construction of habits such as sleeping and eating. A study that used the HPM with Egyptian female adolescents showed that the most powerful determinant of breakfast consumption was related to activities such as going to school, which directly and indirectly affected this behavior by reducing perceived barriers^(16)^.

A study with Iranian adolescents showed that perceived barriers and situational influencers, also components of the HPM, are effective in decreasing levels of physical activity among teenage^(17)^. Before the pandemic, these barriers and influencers were related to the excess of school activities or lack of facilities for exercising^(17)^. In the current context, the need for social isolation requires measures such as wearing masks, avoiding group practices and closing schools and clubs, which can inhibit or discourage physical activity.

This can be confirmed in the “*ConVid* Adolescents” survey, which revealed that most Brazilian adolescents (71.5%) adhered to social restriction measures in a complete or intense way, going out only to supermarkets, pharmacies or relatives’ homes^(5)^.

Thus, the analysis of psychological factors revealed in the questionnaire and in the workshop showed that the students shared difficult feelings and experiences, and conformed the therapeutic potential of the workshop, as they were able to share and recognize the experiences of their peers, as well as compare the educational moment of the workshop with other unsatisfactory virtual experiences restricted to pedagogical activities.

For Paulo Freire, dialogue is the essence of true education, because it is through dialogue that the world is signified and transformed, and it is only possible among the actors involved in a context that allows a horizontal and intentional construction of knowledge^(12)^. This way, conceived by the word in the act of pronouncing the world, authentic dialogue is possible and must be stimulated even in remote teaching, mainly because it reflects the contemporary reality of technologies, adding knowledge both for students^(18-19)^ and for teachers, who also face challenges in this context^(20)^.

Pender highlighted, even before the pandemic, the importance of using technology to promote health, as it has been increasingly present in the daily lives of adolescents^(8)^. Thus, social networks, for example, can help dealing with internal and external barriers. Furthermore, the effects of the experiences mediated by technologies on the neurological development and human interaction will only be seen in the long term^(21)^.

In the meantime, social factors stand out, as the students described the challenges experience with the restriction of social contacts and shared family experiences. Pender proposes instructive strategies so that the people in the daily life of children and adolescents can provide them with social support, whether of informative, emotional, evaluation or instrumental nature, and discusses the frequency and type of contact of those who can be sources of support^(8)^.

It is valid to say that human beings are inherently social. In adolescence, which is a period of psychosocial transition between childhood and adulthood, the school is one of the most important social environments. The concerning situation that many children and adolescents find themselves in when deprived of this context is evident^(21)^. Regarding the challenges, many difficulties of remote education were pointed out: 59% reported lack of concentration, 38.3% lack of interaction with teachers and 31.3% lack of interaction with friends^(5)^.

However, this psychosocial impact is still poorly recognized by society and by adolescents themselves. This can be observed when the students express the losses of not being in school just considering the deficit in knowledge and content assimilation, which is also an issue that cannot be neglected.

Regarding Prior Behavior, the perception of health associated to the possibilities of entertainment during social isolation or in comparison with the experience before the pandemic goes back to the conceptualization of the essence of the object *health* through a social-historical dialectic articulation, as well as the production of health as the production of life itself and its subjectivities^(22)^.

Pender highlights that individuals’ perceptions and interpretations of what they experience directly affect their behavior^(8)^, so it is important to clarify the value and meaning of health before developing a change plan. In the collective scope, Paulo Freire supports that the sharing of reality allows the awareness of the confrontations experienced, the “limit situations”, which sometimes go unnoticed or are seen as insurmountable barriers and, through dialogue, as an effective meeting of people to be more, it is possible to discover unprecedented possibilities, based on which action can be defined^(12)^.

In the aforementioned Brazilian survey, 30% of the participants reported worsening of their health condition during the pandemic^(5)^. Therefore, knowing the strategies that adolescents identify as healthy is important, since these represent strengths to be used to implement educational interventions, favoring access and adherence of young people to what is proposed in the school environment^(8)^.

Based on the analysis of several studies, authors corroborate that negative impacts such as those reported are expected in the short term in the experience of social isolation, and that it is urgent to act in order to mitigate them, as they can have long-term effects, with neurological, psychiatric, psychological and hormonal impairments and their correlations, fundamental for those who are in a phase of full development of these functions, the adolescents^(23)^.

In the Dramatic Action phase, in which the students developed their proposals as candidates for Minister of Health, it was possible to give a voice to these social actors, who had not had their needs heard. Empowerment, made possible by the expression of the imaginary, favors coping and health promotion, as both are based on the assumption that individuals have the ability to generalize, plan and promote change^(8,13)^.

In this moment, the speeches that stood out referred to vaccines and quality of life, in contrast to the Infodemic scenario, which consists of an overabundance of information, some accurate and some not, during an epidemic^(23)^, and moving towards the demystification of reality, which corresponds to the critical understanding^(12)^. According to Freire, oppressors are interested in mythologizing the world to maintain domination; however, no one can unveil the world to the other^(12)^. Therefore, health promotion is enhanced in dialogic relationships, as they allow breaking these myths with the subjects themselves.

The HPM is an important tool for promoting the health of children and adolescents in the context of the pandemic, mainly because it proposes to guide, identify and measure results influenced by nursing actions, at the individual, family or community level, as well as in the short, medium or long term^(8)^. The findings confirm the conclusions of other studies that assessed this intervention and found significant improvements in healthy habits and body indexes in comparison with control groups that did not use the HPM^(24-25)^.

### Study limitations

As limitations of this research, it is possible to point out the selection of a portion of the population, formed by students who had resources such as a computer and internet, marking the perspectives of a socioeconomic context that is distant from the reality of many Brazilian adolescents.

The virtual character of the experience also restricts the observation of body language. In these situations, it is important for researchers/educators to be aware of other types of language and forms of interaction, as well as epistemological silences and their meanings, represented by the lack of interaction, closed camera or loss of connection, which can be intentional.

### Contributions to the area of Nursing

This research confirms that the HPM is a tool that provides nurses with a practice oriented by the health situation and a survey of aspects that are fundamental for health promotion. Thus, it is possible to develop these aspects through participatory strategies and technologies, which favor group engagement and the sharing of experiences in the moments planned.

It contributes to the scientific community by showing the perspectives of adolescents in the context of the pandemic, which should be the focus of actions aimed at adolescent health. It also demonstrates the importance of School Nursing as a space for professional action decisive for health promotion.

## FINAL CONSIDERATIONS

It was possible to construct knowledge about the experiences and health perspectives of adolescents in the face of the Covid-19 pandemic. The theme of mental health can be conceived as a protagonist in the workshop, being part of the reality of adolescents in the condition of social distancing. In addition, it is worth noting that the workshop itself, being by its nature a collective experience, and therefore a therapeutic one, allowed an encounter beyond school routines and the recognition of each singular story identified as collective.

As a complement to the present investigation, studies and participatory programs with a view to welcoming and mental health should be conducted, also considering the many possibilities of the HPM, emancipatory references and information and communication technologies.
